# Clinicopathological Features of Sessile Serrated Polyps in China: A Retrospective Study of a Tertiary Hospital

**DOI:** 10.5152/tjg.2022.22027

**Published:** 2023-02-01

**Authors:** Zhi-Jie Wang, Cheng Luo, Lin-lin Zhao, Shi-yu Li, Hong-yu Fu, Xiao-ju Su, Zhao-Shen Li

**Affiliations:** 1 Department of Gastroenterology, Changhai Hospital, Second Military Medical University/Naval Medical University, Shanghai, China; 2 Department of Pediatric Surgery, Women’s Hospital of Nanjing Medical University, Nanjing Maternity and Child Health Care Hospital, Nanjing, China

**Keywords:** Advanced adenomas, detection rate, serrated polyp

## Abstract

**Background:**

Serrated polyps have been recognized as the important premalignant lesions. In this study, we aimed to analyze the clinicopathological features of sessile serrated polyps and determine the association between sessile serrated polyps and synchronous advanced adenomas.

**Methods:**

Consecutive patients undergoing diagnostic or therapeutic colonoscopies (including 156 681 diagnostic colonoscopies) from 2011 to 2019 were included.

**Results:**

A total of 958 patients, including 699 (73%) males, were detected with at least 1 sessile serrated polyp, and 65.9% (n = 658) of sessile serrated polyps were located in the distal colon. Advanced serrated lesions accounted for 9.1% (n = 91) of all the sessile serrated polyp (n = 999). The types of SSP included flat type (953/999, 95.4%) and sub-pedunculated or pedunculated type (46/999, 4.6%). Meanwhile, there was no obvious evidence supporting the association between advanced adenomas and characteristics of advanced serrated lesions or sessile serrated polyps.

**Conclusion: S:**

essile serrated polyps seem to be more frequently seen in the distal colon of men in this study. However, more evidence is required to confirm the actual distribution of sessile serrated polyp in colon among Chinese people. There is still much room for improvement of sessile serrated polyp detection rate, and more importance should be attached to sessile serrated polyp both for pathologists and endoscopists.

**Main Points**

- Among 156 681 diagnostic colonoscopies in our study, 0.36% (562/156 681) had at least 1 sessile serrated polyps (SSPs), and SSPs seem to be more frequently seen in the distal colon of men in China.
- Bivariate analyses revealed that a higher proportion of physicians had an above-median sessile serrated polyp detection rate (SSPDR) than that of surgeons (77.8% vs 0%; *P*<.01).
- There was no obvious evidence supporting the association between advanced adenomas and characteristics of advanced serrated lesions or SSP.
- The mean SSPDR was 0.70% (±0.68%; range, 0%-2.8%) across endoscopists. Thus, there is still much room for improvement of SSPDR, and more importance should be attached to SSPs both for pathologists and endoscopists.

## Introduction

In addition to the sequence from adenoma to carcinoma, colorectal cancer (CRC) also occurs via the serrated pathway.^1,2^ Over the last decade, serrated polyps have been recognized as the important precancerous lesions, which might account for up to 30% of CRC cases.^3^ Serrated polyp can be divided into hyperplastic polyp (HP), sessile serrated polyp (SSP, also known as sessile serrated adenoma or -lesion), and traditional serrated adenoma (TSA). Of them, SSP and TSA are recommended to be removed for their malignant potentials. Traditional serrated adenoma is reported to represent about 1% of serrated polyps, while SSP takes up approximately 20%.^4-9^ Thus, SSP is the most common and important type of precancerous serrated polyp. However, it is difficult to detect SSP by screening tests such as fecal immunochemical testing and computed tomography colonography.^10^ Besides, compared with traditional adenomas, the endoscopic visualization of SSP is poor due to its subtle appearance and typically appears as flat or sessile with indistinct margins. Previous studies have demonstrated a significant variation in serrated polyp detection rate across endoscopists and other factors, though some determinants of this variation have been identified.^11-16^ However, most of these studies were conducted in Western countries, while evidence from China is rare. It is proven that the prevalence and clinical features of serrated polyps vary among different regions in the world.^17^ For example, the majority of serrated polyps are reported to be located in the proximal colon,^14,17,18^ but a study in China shows that SSPs are located more in the distal colon.^19^ Considering this, we believe that convincing data from China may contribute to a more comprehensive understanding of the causes of the low detection rate of serrated polyps around the world and that we can tailor our solutions accordingly to improve the colonoscopy quality.

Thus, this retrospective study aimed to analyze the clinicopathological features of SSP in China and determine the association between SSP and synchronous advanced adenomas.

## Materials and Methods

In this single-center, retrospective study, consecutive colonoscopy records from the Endoscopy Center of Changhai Hospital (tertiary hospital) from March 1, 2011, to January 1, 2019, were reviewed. In addition, endoscopy and pathology reports of all the included polyps were also reviewed. Patients who received a prior colonoscopy within 6 months or those with incomplete examination, inflammatory bowel disease, or CRC were excluded. Endoscopists who performed less than 100 colonoscopies during the study period were excluded. This article received an institutional approval waiver by the ethics committee of Changhai Hospital due to the retrospective nature of outcome report.

The patient, endoscopist, and procedure characteristics were collected. Patient characteristics included age, gender, endoscopic records (location, number, and size of polyps), and pathological results. Endoscopist characteristics involved age, gender, specialty, years of practice, and number of colonoscopies performed during the study period. Procedure characteristics included the time of colonoscopy and withdrawal time. Nearly all patients received the split-dose bowel preparation with 2 L or 3 L of polyethylene glycol in our center, as described previously.^20,21^ Bowel preparation using other methods was rare and negligible (accounting for less than 5% according to rough estimation). Boston Bowel Preparation Scale (BBPS) score was used to assess bowel preparation in our center. Generally, we routinely perform polypectomy for outpatient if the size of polyp is ≤0.5 cm, and if the size of polyp is larger than 0.5 cm, we usually perform biopsy and advise the patient to be hospitalized for polypectomy. The pathological diagnosis of biopsy from outpatient would be reconfirmed by the polyp specimen after polypectomy (if available), and World Health Organization (WHO) classification 2010 was applied.

Sessile serrated polyps with dysplasia or diameter ≥ 10 mm and all TSAs were identified as advanced serrated lesions (ASLs).^22^ Additionally, advanced adenomas were defined as adenomas of size ≥1 cm, with high-grade dysplasia or with a villous component. The SSP detection rate (SPPDR) was defined as the proportion of colonoscopies with ≥1 SSP among all diagnostic colonoscopies. Similar to previous study,^14^ SSP and SPPDR in this study also included TSA because TSA is rare (<0.07% in our samples) compared with SSP and has a minimal impact on the SSP and SSPDR measure. Hyperplastic polyp was not included in this study. Proximal colon was defined as colon proximal to the splenic flexure (including cecum, ascending colon, hepatic flexure, and transverse colon), whereas distal colon was defined as colon between the splenic flexure and the anal verge (including splenic flexure, descending colon, sigmoid colon, and rectum).

### Statistical Analysis

Continuous data were summarized as means with standard deviation. Logistic regression analysis was conducted to analyze the odds ratio and 95% CI for synchronous advanced adenomas. Meanwhile, the polyp size between ASL and non-ASL groups was compared by independent sample *t*-test. Categorical data were expressed as frequencies with percentages and compared by chi-square or Fisher’s exact test. Values with 2-sided *P <* .05 were considered statistically significant. All statistical analyses were performed using Statistical Package for the Social Sciences version 21.0 software (IBM Corp.; Armonk, NY, USA).

## Results

### Patient and Endoscopist Characteristics

A total of 958 patients were detected with at least 1 SSP in all the colonoscopies performed in Changhai Hospital from March 1, 2011, to January 1, 2019. Among them, 58.7% (n = 562) were diagnostic colonoscopies (outpatient colonoscopies), while the remaining 41.3% (n = 396) were therapeutic colonoscopies. The mean age of patients was 54.2 (±11.3) years, and 73% (n = 699) of patients were male.

All the colonoscopies were performed by 56 endoscopists of whom 67.9% (n = 38) were male and 35.7% (n = 20) were surgeons. The mean years of practice time following the completion of fellowship was 11.1 (±7.3) (range, 2-40) years. The mean number of diagnostic colonoscopies performed during the study period was 2798 (±3721) (range, 106-19341). Among the diagnostic colonoscopies, the mean withdrawal time (including the time of biopsy) of surgeons and physicians was 6.6 ± 2.5 minutes and 7.9 ± 4.0 minutes, respectively (*P* = .04) (Table 1).

### Sessile Serrated Polyp Characteristics

Among the 958 patients with SSP, 54.9% (n = 526) had multiple polyps and 3.3% (n = 32) had at least 2 SSP. Altogether, 999 SSPs were detected in 958 patients. Advanced serrated lesions accounted for 9.1% (n = 91) of all the SSPs.

In our study, 65.9% (n = 658) of SSPs were located in the distal colon, whereas the remaining 34.1% (n = 341) were located in the proximal colon. Meanwhile, 43.4% (148/341) of SSPs located in the proximal colon were located in the transverse colon. The mean size of polyps was 0.54 (±0.31) cm. The types of SSP included flat type (953/999, 95.4%) and sub-pedunculated or pedunculated type (46/999, 4.6%). The characteristics of SSP (including 6 cases of TSA) are shown in Table 2.

### Sessile Serrated Polyp Detection

During our study, there were 156 681 diagnostic colonoscopies, and 0.36% (562/156 681) had at least 1 SSP. The mean SSPDR was 0.70% (±0.68%; range, 0%-2.8%) across endoscopists. Bivariate analyses revealed that a higher proportion of physicians had an above-median SSPDR than that of surgeons (77.8% vs 0%; *P* < .01). Besides, endoscopists with the lowest procedure volume were more likely to have an above-median SSPDR than endoscopists with a higher-procedure volume (*P* = .027) (Table 3).

**Figure 1.**
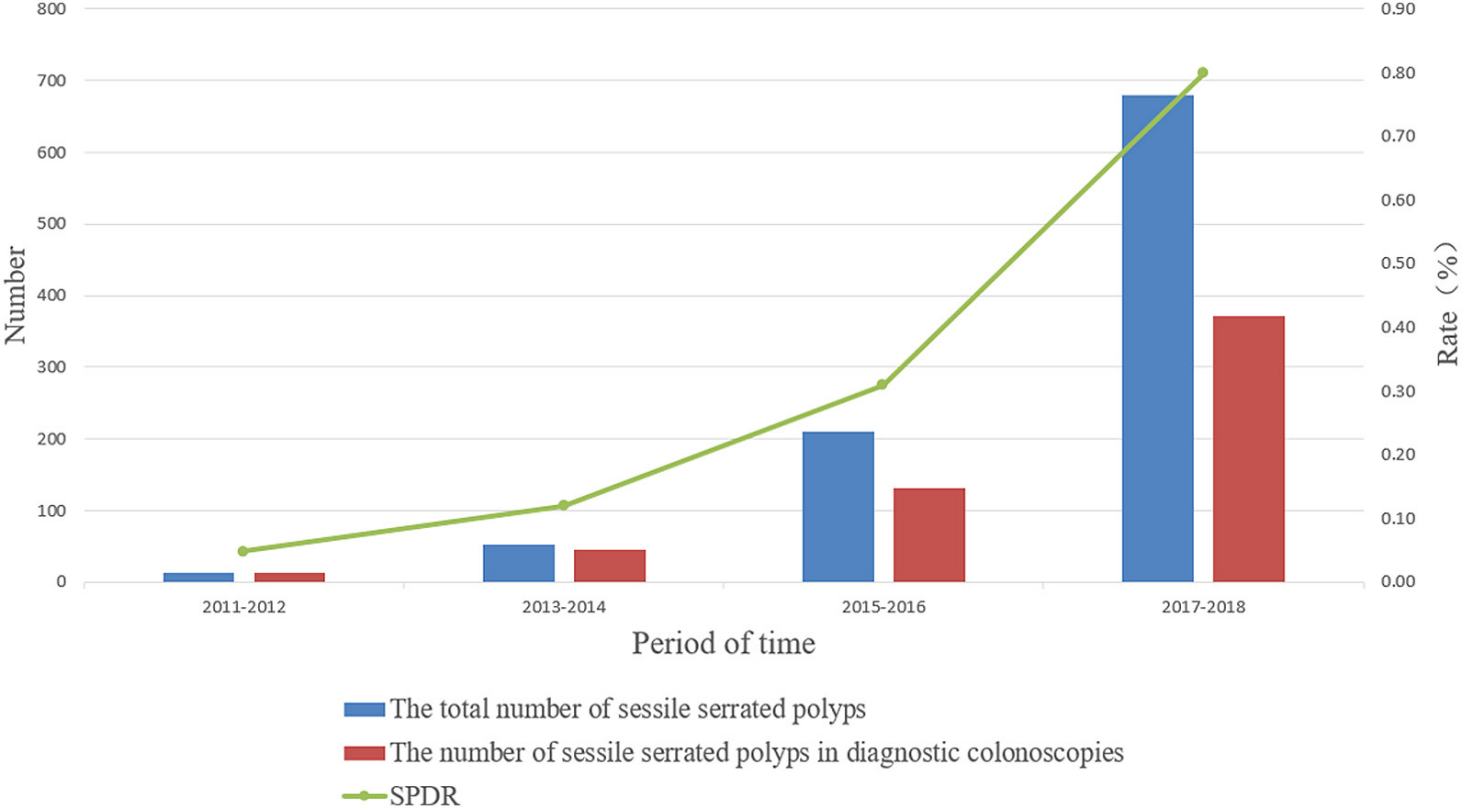
The number of sessile serrated polyps (SSP) detected and the SSP detection rate (SSPDR) in different periods of time.

When stratified by time period, the number of SSPs detected substantially increased year by year. In the meantime, the SSPDR (0.8%) in 2017 and 2018 was elevated by 16 folds compared to that (0.05%) in 2011 and 2012 (Figure 1).

### The Relationship Between Sessile Serrated Polyp and Advanced Adenomas

There were 88 (9.2%) SSP patients with coexisting advanced adenomas. The mean size of advanced adenomas was 0.97 (±0.73) cm. In addition, 56.8% (n = 50) of advanced adenomas were located in the distal colon, whereas the rest were located in the proximal colon. Of the 91 ASLs, there were 22 patients with coexisting advanced adenomas (Table 4). Neither univariate nor logistic regression analysis revealed any significant results.

## Discussion

Although several studies have discussed the clinical and pathological features of colorectal serrated lesions,^23-25^ evidence from Asia, especially from China, is small and insufficient. In this study, we detected 958 patients with SSP from over 156 681 colonoscopies performed by 56 endoscopists. We focused on the clinicopathological features of SSP, ASLs, the association between ASLs and synchronous advanced adenomas, and the endoscopist characteristics associated with SSPDR above and below the median SSPDR. According to our results, 30.5% of SSP and 24.2% of ASLs were detected in patients aged under 50 years, reminding us of the risk of CRC in middle-aged population. The present study also showed that 73% of patients were male and 65.9% of SSPs were located in the distal colon. The location distribution of SSP is not consistent with previous reports, and it should be investigated carefully in well-designed prospective studies among Chinese people in the future. Areal variation, different age distribution, poorer bowel preparation in the proximal colon, or other factors may contribute to this result in current study. Meanwhile, only 4.6% of SSPs belonged to the sub-pedunculated or pedunculated type. Therefore, endoscopists must be aware of these flat polyps to improve their detection.

Similar to other studies conducted in China,^23,25^ the mean SSPDR across endoscopists is quite low compared to the data reported from Western countries.^14,26^ Several reasons may be responsible for this, as shown below. (1) Some serrated polyps may be misclassified or misdiagnosed by pathologists due to the confusing nomenclature and changing pathological criteria in the past. (2) Inadequate attention is paid to the endoscopic diagnosis of serrated polyps. (3) The ability to detect these flat polyps with indistinct margins is low, since narrow-band imaging or high-definition endoscopy equipment is not always available several years ago. (4) In our center, some endoscopists would not always perform biopsy during outpatient colonoscopies when the patients need to be hospitalized for polypectomy in the early years.

Serrated polyps are classified as HP, SSP, and TSA according to the WHO classification in 2010. Since then, endoscopists and pathologists in China have paid more attention to these precancerous polyps. This is also reflected in our study that the number of SSP detected increased from 14 (2011-2012) to 680 (2017-2018), and the SSPDR was 16 times higher (2011-2012 vs. 2017-2018). Therefore, it is also not difficult to understand why endoscopists with lower procedure volume were more likely to have an above-median SSPDR unexpectedly in our study. Most of the endoscopists with a lower procedure volume tend to perform colonoscopy more recently.

When it comes to endoscopist factors associated with the above-median SSPDR, bivariate analyses revealed that the performance of physicians was much better than surgeons. Typically, withdrawal time was the most probable cause. In our study, the mean withdrawal time of surgeons was significantly shorter than that of physicians. The longer withdrawal time has been proven to be associated with betterserrated polyp detection.^13,27^ Moreover, increasing emphasis has been laid on the withdrawal time among physicians in our center in recent years.^28^ Thus, the importance of withdrawal time should be repeatedly emphasized among surgeons. Meanwhile, differences in biopsy strategy towards outpatients may also account for the lower SSPDR among surgeons during diagnostic colonoscopies. Additionally, we tried to identify the relationship between SSP or ASL and synchronous advanced adenomas but failed to find any significant difference. Similar issue had been discussed in a study from Korea.^29^

Several limitations should be noted in our study. First, this was a single-center retrospective study, which might have selection bias. Second, we failed to conduct logistic regression at the colonoscopy level to identify the endoscopist and procedure factors associated with serrated polyp detection because the number of negative cases (n = 15 119) was too bulky to be processed manually. Third, the BBPS score of all the patients in our current study is hard to be calculated because there are 156 681 diagnostic colonoscopies. However, it is estimated that patients with BBPS score ≥6 accounts for about 80% of all the patients according to previous studies in our center.^21,30^ Fourth, there is a small number of patients who may refuse polypectomy (biopsy) or hospitalization in our center. Although the number is quite small, it will actually affect the results of the study. The actual SSSPDR should be slightly higher than described in this study.

In summary, knowledge of serrated polyps has been disseminated slowly, and inadequate importance is attached among pathologists and endoscopists in China. More evidence is required to confirm the actual distribution of SSP in colon among Chinese people. Considering the indistinct margins and very flat morphology of serrated polyps, the endoscopic diagnostic skill and new techniques like narrow-band imaging,^31,32^ blue laser imaging,^33^ and artificial intelligence^34^ should be applied widely. Overall, there is much room for the improvement in SSP detection.

## Figures and Tables

**Table 1.** Characteristics of Patients with Sessile Serrated Polyps (SSP) and Procedure

Charactertistic	Overall, n(%)
Patient demographics	
Total	958
Sex	
Female	699 (73)
Male	259 (27)
Mean age, year (SD)	54.2 (±11.3)
Procedure information	
Indication	
Diagnostic colonoscopies	562 (58.7)
Therapeutic colonoscopies	396 (41.3)
Mean withdrawal time, min (SD)	7.8 (±3.9)
Surgeons	6.6 (±2.5)
Physicians	7.9 (±4.0)

SD, standard deviation.

**Table 2.** Characteristics of Sessile Serrated Polyps (SSP)

	SSP			Non-ASL	ASL	*P*
	SSP(Total)	SSP(SSP)	SSP (TSA)			
Sex						
Female	259	258	1	236	23	.691
Male	699	694	5	631	68	
Age						
≥50	666	662	4	597	69	.170
<50	292	290	2	270	22	
Size	0.54 (±0.31)			0.47(±0.13)	1.31(±0.87)	<.001
≥1 cm	76	76	0	0	76	<.001
<1 cm	923	917	6	908	15	
Location						
Distal	658	657	1	616	42	<.001
Proximal	341	336	5	292	49	
Morphology						
Flat	953	947	6	881	72	<.001
Non-flat	46	46	0	27	19	

TSA, traditional serrated adenoma;

ASL, advanced serrated lesions; SSP, sessile serrated polyps.

**Table 3.** Endoscopist Factors Associated with Sessile Serrated Polyp Detection Rates (SSPDR) Above and Below the Median Rate

	Overall, n (%)	Below Median SSPDR, n (%)	Above Median SSPDR, n (%)	*P*
Total endoscopists	56(100)	28(50)	28(50)	-
Sex				
Female	18(32.1)	4(22.2)	14(77.8)	.622
Male	38(67.9)	24(63.2)	14(36.8)	
Specialty				
Physician	36(64.3)	8(22.2)	28(77.8)	<.001
Surgeon	20(35.7)	20(100)	0(0)	
Practicing time, year				
≤5	9(16.1)	6(66.7)	3(33.3)	.469
6-10	25(44.6)	6(24)	19(76)	
11-15	11(19.6)	7(63.6)	4(36.4)	
≥16	11(19.6)	9(81.8)	2(18.2)	
Procedure volume during the study period				
100-500	10(17.9)	0(0)	10(100)	.027
501-1000	10(17.9)	5(50)	5(50)	
1001-2000	14(25.0)	8(57.1)	6(42.9)	
2001-4000	11(19.6)	7(63.6)	4(36.4)	
≥4001	11(19.6)	8(72.7)	3(27.3)	

SSPDR, sessile serrated polyp detection rates.

**Table 4.** The Relationship Between Advanced Serrated Lesions and Synchronous Advanced Adenomas

	ASL With Advanced Adenomas	ASL Without Advanced Adenomas	*P*
Sex			
Female	6	17	.804
Male	16	52	
Age			
≥50	15	54	.336
<50	7	15	
Size			
≥1 cm	21	55	.161
<1 cm	1	14	
Location			
Distal	13	28	.129
Proximal	9	41	
Morphology			
Flat	18	54	.955
Non-flat	4	15	

ASL, advanced serrated lesions.
